# Sustained release of amoxicillin from hydroxyapatite nanocomposite for bone infections

**DOI:** 10.1007/s40204-018-0103-4

**Published:** 2018-11-27

**Authors:** A. P. S. Prasanna, G. Devanand Venkatasubbu

**Affiliations:** 1 Department of Physics, SRM Institute of Science and Technology, Kattankulathur, Kanchipuram, Tamil Nadu India; 2 Department of Nanotechnology, SRM Institute of Science and Technology, Kattankulathur, Kanchipuram, Tamil Nadu 603 203 India

**Keywords:** Hydroxyapatite, Polyvinyl alcohol, Sodium alginate, Amoxicillin, Antimicrobial studies

## Abstract

Hydroxyapatite (HAP) is the main constituent of human bone and teeth. Hydroxyapatite nanoparticles are used for the treatment of various bone infections. Nanohydroxyapatite is a biocompatible material. It is used as a drug carrier for drugs and biomolecules for various diseases. Hydroxyapatite nanoparticles are made into nanocomposite with sodium alginate and polyvinyl alcohol. This nanocomposite is used for the sustained release of drugs. It is characterized by various characterization techniques like XRD, FTIR, TEM, and Raman. Hydroxyapatite nanoparticles are coated initially with polyvinyl alcohol and then coated with sodium alginate. Amoxicillin is used as the model drug. Studies on the drug loading and drug release have been done. The release of the drug is sustained for about 30 days. Antimicrobial studies have shown good activity against pathogens. The zone of inhibition is found to be 18 mm for a concentration of 500 µg against *Bacillus subtilis* and 16 µg against *Klebsiella pneumonia.*

## Introduction

Sustained release formulations have several advantages over traditional drug delivery systems. In sustained drug delivery, drug is released at a predetermined rate for a longer period of time. In traditional drug delivery methods, drug is distributed throughout the body. But in sustained drug delivery, drug is released at the local site of infection. This leads to increased therapeutic index and therapeutic efficacy. This reduces serum concentration and side effects on other organs. Drug stability, optimized drug absorption and prolonged drug release can be achieved by localized drug delivery. Drug carrier is an important part of drug delivery system. It incorporates the drug, retains it and releases it progressively with time. Therefore, properties like drug incorporation and release, formulation stability and shelf life, biocompatibility and biodistribution, and functionality should be analyzed thoroughly when choosing a carrier for delivery of drugs. The drug release from any carrier depends on the solubility of the drugs, microstructure of carrier, degradation of carrier and the bond between the drug and the carrier (Devanand Venkatasubbu et al. 2011).

Bone infections like osteomyelitis are major challenges in treating bone diseases. Bone infections are majorly caused by bacterial infections. Immediately after infection, the bacteria form biofilms. The bacteria form clusters and they are attached to the extracellular matrix (ECM). Due to this process, they are protected from the body’s immune system. The antibiotics used to treat such bone infections are also not able to reach the infected site due to the presence of bioflim. All these make bone infections most difficult to treat. Such bone infections can only be treated with sustained drug delivery systems which deliver antibiotics at the infection site. Nanocarriers which are biocompatible and biodegradable are used for the sustained delivery of antibiotics for none infections. Bone infections are treated with biocompatible nanomaterial like hydroxyapatite. The nanomaterials have the ability to carry the drug molecules on its surface. The porous nature of these nanomaterials makes them a suitable material for the sustained release of drug molecules. The porosity helps in the sustained release of drug and in the reconstruction of bone (Parent et al. 2016; Gomes et al. 2013; Yan et al. 2015)

Hydroxyapatite (HAP) (Ca_10_(PO_4_)_6_(OH)_2_) is used for the preparation of drug delivery system. It is used for the sustained release of various drugs and biomolecules because of its excellent properties, such as the ability to adsorb a variety of chemical species and biocompatibility (Andrés et al. 2018). However, the release of drugs from HAP has been proved to be initially very fast, owing to the weak interaction between the drugs and the HAP particles (Mizushima et al. 2006). HAP nanoparticles when combined with polymer will prolong the release of drugs to make the composites applicable for long-term sustained release. HAP nanoparticles used in drug delivery systems should be in submicron range when implanted in the body. The drug loading capacity should be high. It is non toxic (Devanand Venkatasubbu et al. 2015; Devanand Venkatasubbu et al. 2013a; Miculescu et al. 2017). There are no side effects. Nanosized hydroxyapatite, when used as a carrier for the delivery of drug and other therapeutic agents, enhances bioavailability, predictable therapeutic response, greater efficacy and safety, sustained and prolonged release. The usage of hydroxyapatite for drug delivery is effective (Devanand Venkatasubbu et al. 2012).

HAP nanoparticles are used as an effective non-viral vector for gene delivery (Bisht et al. 2005). The polymer hydroxyapatite nanocomposites are very much used for the treatment of bone disorders and infections. HAP drug compositions have been used as a bone substitute for the delivery of anticancer drugs (Palazzo et al. 2007; Barroug and Glimcher 2002). Polymer/HAP is used in sustained release of drug molecules, growth factors for various bone disorders and as scaffolds for cell growth (Wei and Ma 2004; Huang et al. 2008). PLGA/HAP composite has been prepared by electrospinning. It is used for the sustained release of amoxicillin for bone infection (Zheng et al. 2013). HAP/PLGA microspheres are also used for the release of alendronate for bone disorders (Shi et al. 2009).

Alginates are a family of unbranched binary copolymers. Alginates are natural polysaccharide polymers isolated from brown seaweed. Alginates with monovalent ions are generally water soluble, while salts with bivalent cations like Ca^2+^ form insoluble hydrogels. In the case of partial binding with bivalent ions, solubility in water is partially retained. Calcium cross-linked alginate hydrogels have been used in many biomedical applications, including cell transplantation and drug delivery. Alginates are biocompatible and biodegradable polymers. They are widely used in many biomedical applications. They are used as carrier for drugs and biomolecules. They are used as scaffolds for tissue engineering (Ribeiro et al. 2004).

Polyvinyl alcohol (PVA), which is made from polyvinyl acetate through hydrolysis, is easily degradable. It has been applied in the industrial, commercial, medical, and food sectors. PVA remains one of the widely used polymer group of biomaterials applied for medical implants. This usage is due to its segmented block copolymer structure. Due to this wide range of versatility, they are used in applications such as tissue scaffolding, artificial cartilage and biodegradable scaffolds (Rajkumar et al. 2010). PVA is a biodegradable polymer and its degradability is enhanced through hydrolysis because of the presence of hydroxyl groups. Moreover, it is water soluble and has a hydrophilic nature (Qiu and Netravali 2013). Amoxicillin (α-aminohydroxybenzylpenicillin) is a semi-synthetic antibiotic, belonging to the β-lactam family, which is effective against bacterial infections. It is a broad-spectrum antibiotic. Amoxicillin acts by inhibiting bacterial cell wall synthesis. Amoxicillin is susceptible to beta-lacatamase degradation.

In this study, we have synthesized hydroxyapatite nanoparticles. The hydroxyapatite nanoparticles are used as nanocarriers for the sustained release of amoxicillin for treatment of bone infections. The drug release is sustained by coating the hydroxyapatite nanoparticles with biocompatible polymers. Coating the nanocarriers with polymers, it would sustain the drug release. The polymeric coating is done layer by layer to have more sustained release of drug. The nanoparticles are coated with polyvinyl alcohol and sodium alginate in a layer-by-layer method. HAP/PVA/SA nanocomposite is used for the sustained release of amoxicillin for bone infection. The layer-by-layer coating of polymers on the hydroxyapatite nanoparticles leads to the sustained release of drug. Drug loading and drug release studies have been done. A sustained release of amoxicillin is observed.

## Experimental

### Synthesis and characterization of hydroxyapatite nanoparticles

Orthophosphoric acid (0.6 M) was added to calcium hydroxide (1 M). It was stirred for 2 h at room temperature. The pH of the solution was maintained at 11. The sample was washed repeatedly and dried at 80 °C (Devanand Venkatasubbu et al. 2011).

### Synthesis of hydroxyapatite/polyvinyl alcohol composite

Polyvinyl alcohol at 2.5 g was dissolved in 50 mL of water. Hydroxyapatite nanoparticles were added to the solution and stirred for 2 h. The precipitate was separated by centrifugation and dried at room temperature.

Powder X-ray diffraction (XRD, Seifert, JSO-DE BYEFLEX 2002, Germany) was utilized for X-ray diffraction analysis. The size of the nanoparticle was measured by transmission electron microscopy (TEM). The instrument was Jeol 2000Fx-II operated at 200 kV, high resolution, analytical TEM with a W-source and a point–point resolution of 2 Å. The SEM image was taken with SEC Desktop mini SEM SNE3200 M. The functional groups present in the hydroxyapatite were analyzed by a Perkin Elmer FTIR (spectrum 1). The micro-Raman scattering experiments were carried out using The LabRam HR 800 micro-Raman Spectrometer having 632 nm line of the He–Ne laser as excitation source having 17 mW power.

### Synthesis of hydroxyapatite/polyvinyl alcohol/sodium alginate/amoxicillin nanocomposite

Sodium alginate of 2.5 g was added to 50 mL of water with stirring. Polyvinyl alcohol-coated hydroxyapatite nanoparticles were added to the solution and stirred. Amoxicillin of 2.5 g was added concurrently to the solution and stirred for 2 h. The sample was separated by centrifugation and dried at room temperature. The drug entrapment efficiency was calculated spectrophotometrically at 231 nm wavelength:

1 $${\text{Drug}}\;{\text{entrapment}}\;{\text{efficiency}}\;(\% ) = \left[ {\left( {X - Y} \right)/X} \right] \times 100,$$

where *X* and *Y* are the initial and final drug concentrations

The drug entrapment efficiency is calculated using a standard graph for amoxicillin. Amoxicillin drug solution is prepared at different concentrations by serial dilution method. A standard graph is by taking OD at 231 nm. This standard graph is used to calculate the drug entrapment efficiency.

### Drug release: an in vitro study

One hundred milligram of the sample was added into 100 mL of phosphate-buffered saline (PBS) in a glass bottle at 37 °C at pH 7.4. The drug release was analyzed for 30 days. Sample of 5 mL was withdrawn at constant time interval. The withdrawn buffer was replaced immediately with 5 mL of fresh PBS medium. Amoxicillin concentration in the collected samples was measured at 231 nm spectrophotometrically.

### Antibacterial activity of nanocomposite

The antibacterial activity of the nanocomposite was analyzed against *Bacillus subtilis, Klebsiella pneumoniae* using agar well diffusion method (Malibari 1991; Zhou et al. 2006; Gong and Guo 2009; Zhang et al. 2009). The nanocomposite was taken at different concentrations (50 µg, 100 µg, 200 µg and 500 µg/well). *Bacillus subtilis* and *Klebsiella pneumoniae* are inoculated in nutrient agar plates. The wells were made in plates with a cork borer. The plates were incubated for 24 h at 37 °C. The formation of inhibition zone around the well was measured.

## Results and discussions

**Fig. 1:**
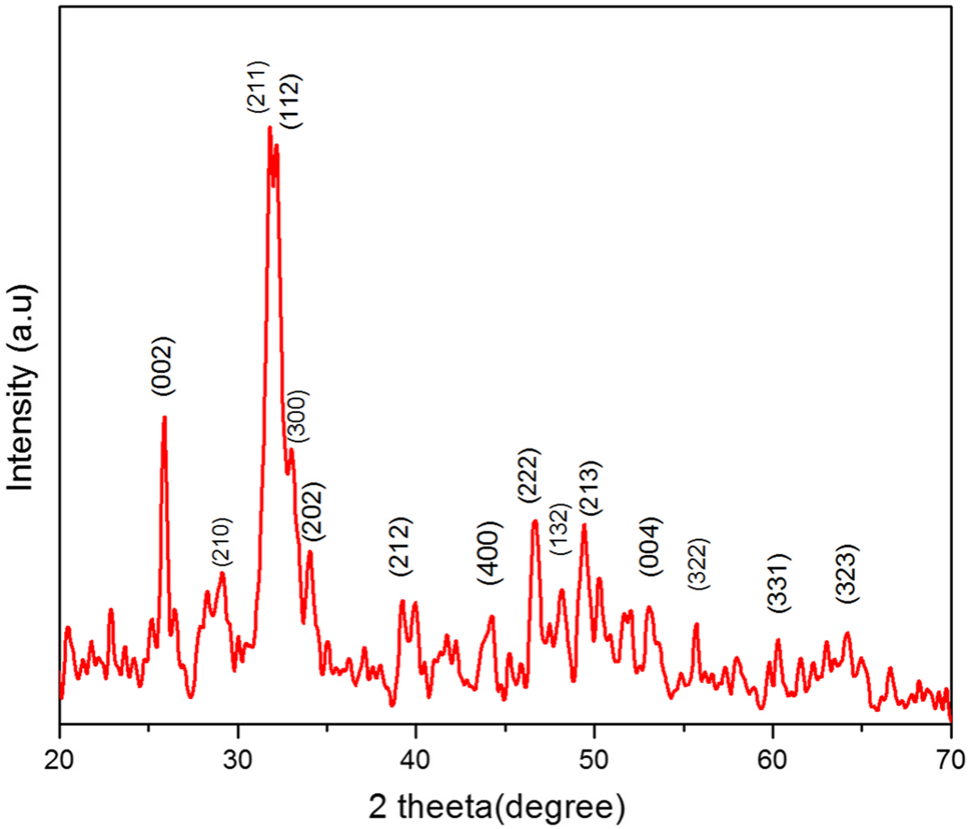
XRD of hydroxyapatite nanoparticles

The HAP nanoparticles and polymer/HAP nanocomposites synthesized were characterized by various techniques. The structure, vibrational mode and functional groups present in the nanoparticles were identified in polymer nanocomposite. The X-ray diffraction image of hydroxyapatite nanoparticle is given in Fig. 1. The XRD image confirms the formation of pure hydroxyapatite nanoparticles. The nanoparticles are crystalline in nature. It matches well with the standard data (JCPDS 09-0432). Peaks representing diffractions (200), (111), (002), (211), (112), (300), (202), (310), (222) and (213) confirm the formation of hydroxyapatite nanoparticles. The peak broadening confirms that the particles are in nanosize. The XRD results confirm that the nanoparticles are suitable to be used as a drug carrier due to their nanosize dimensions.

**Fig. 2:**
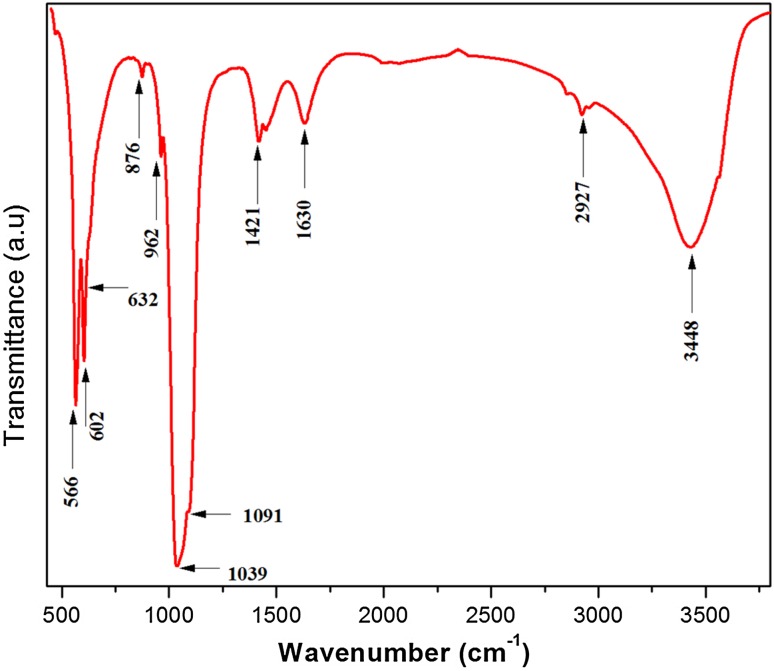
FTIR of hydroxyapatite nanoparticles

Figure 2 shows the FTIR spectrum of HAP nanoparticles. The bands at 1091 and 1039 cm^−1^ are due to *υ*_3_ vibrational mode of phosphate group. The bands at 962 cm ^−1^ and 473 cm^−1^ are due to the *υ*_1_ and *υ*_2_ vibrational modes of phosphate group. The bands at 602 cm^−1^ and 566 cm^−1^ represent *υ*_4_ vibrational band of phosphate group. The peak at 3564 cm^−1^ is because of OH stretching of HAP. The OH bending vibration of the absorbed water is seen at 1630 cm^−1^. The FTIR analyses are in agreement with the XRD results. They confirm that the nanoparticles are pure and there is no impurity (Devanand Venkatasubbu et al. 2013b).

**Fig. 3:**
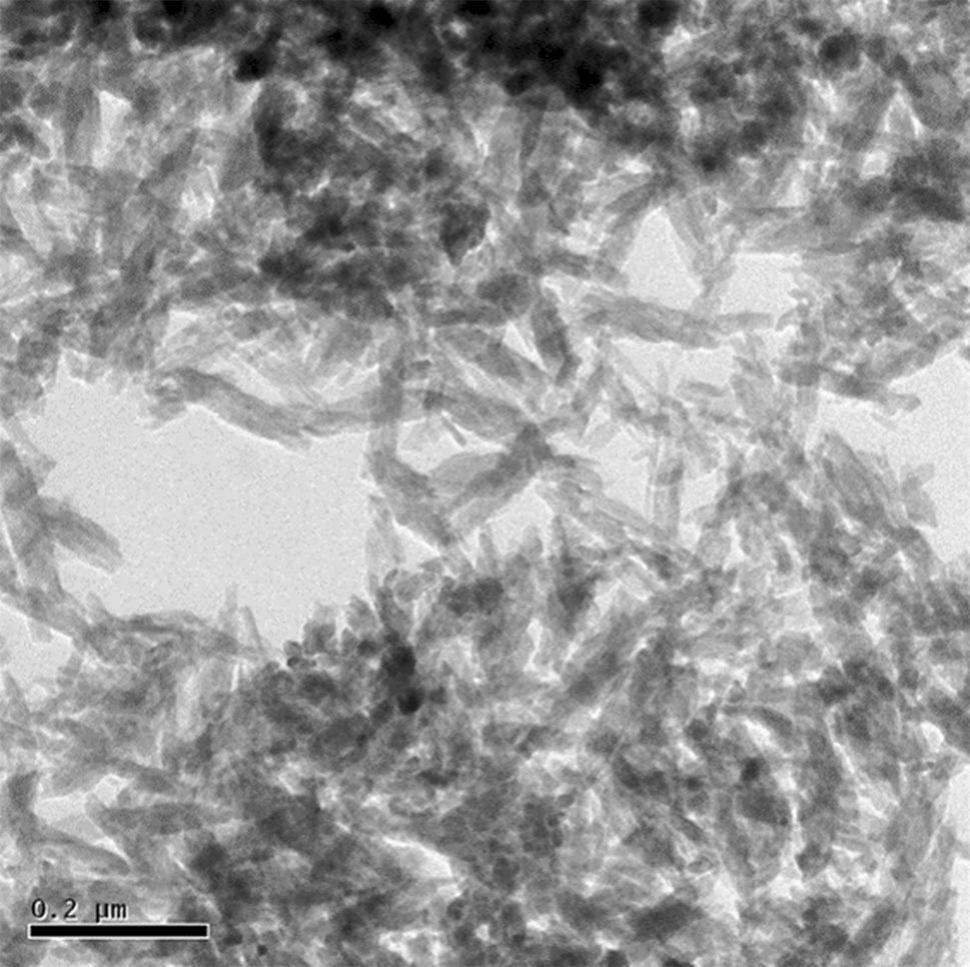
TEM of hydroxyapatite nanoparticles

TEM image of the HAP nanoparticle is given in Fig. 3. The TEM analysis is in agreement with the XRD results. It confirms the nanoparticle size of HAP formation. Needle-shaped HAP nanoparticles are seen in TEM image. The size of the particles is found uniform. They are agglomerated. The length of the particle is about 60 nm and the diameter is 15–25 nm. The edges of the particles are found to be sharp.

**Fig. 4:**
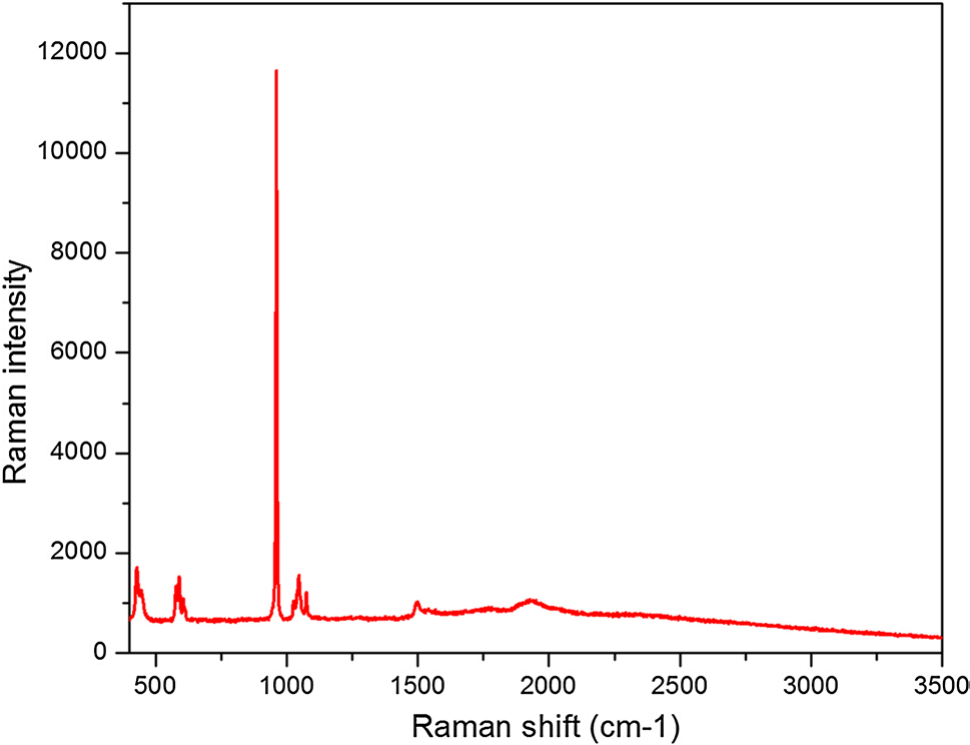
Raman spectrum of hydroxyapatite nanoparticles

Figure 4 represents the Raman image of HAP. The spectrum confirms that the particles formed are hydroxyapatite. The bands observed represent various vibrational modes of hydroxyapatite. The symmetric stretching mode of PO_4_^3−^ ion is found at 968 cm^−1^. The bands at 434 and 489 cm^−1^ represent *υ*_2_ mode of PO_4_^3−^. The band at 594 cm^−1^ is because of *υ*_4_ vibration of PO_4_^3−^ ion. The bands at 1058 and 1084 cm^−1^ are due to *υ*_3_ vibrations of PO_4_^3−^ ion. The phase purity of the HAP nanoparticles is confirmed by Raman analysis (Devanand Venkatasubbu et al. 2011).

The drug loading percentage was calculated by plotting the standard graph. The drug loading percentage was found to be 46%. The drug-loaded samples were analyzed by FTIR spectroscopy to confirm the polymer coating and drug loading.

**Fig. 5:**
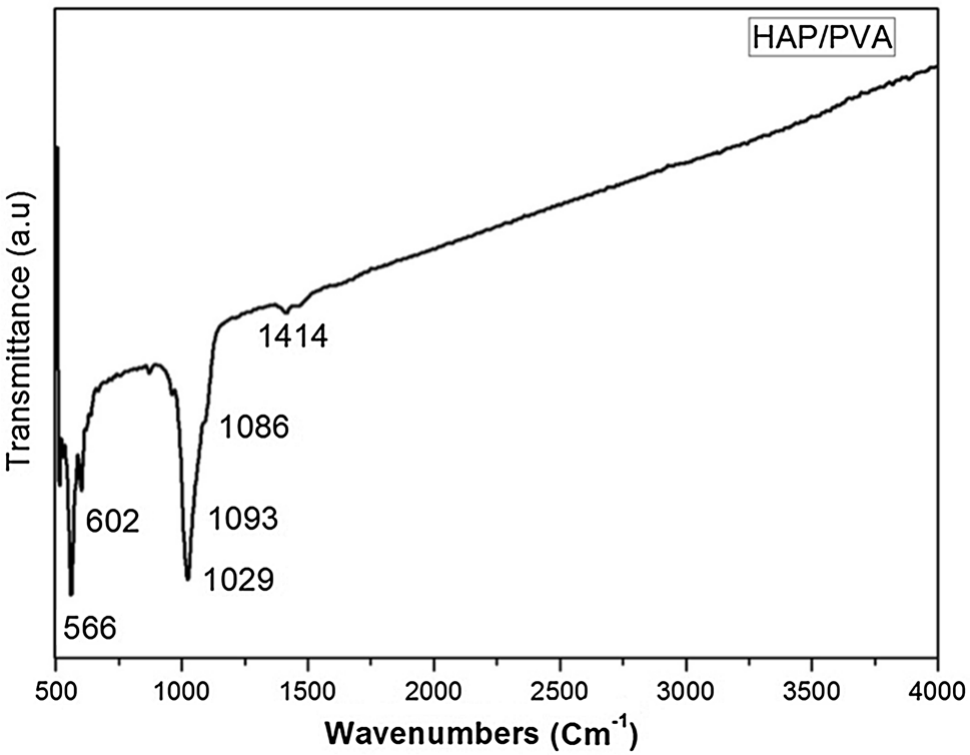
FTIR spectrum of HAP/PVA nanocomposite

The FTIR spectrum of pure PVA clearly reveals the major peaks associated with polyvinyl alcohol. The band around 3600–3650 cm^−1^ is due to the hydroxyl band of free alcohol. The peak at 1142 cm^−1^ represents the structure of polyvinyl alcohol. The peak around 2830–2695 cm^−1^ is due to the C–H bond of aldehyde. The band at 2840–3000 cm^−1^ is because of the C–H vibration of alkyl groups, the band at 1414 cm^−1^ represents –C–O group and 1150–1085 cm^−1^ is because of the vibration of C–O–C (Devanand Venkatasubbu and Anusuya 2017). The FTIR spectrum of HAP/PVA composite is given in Fig. 5. The spectrum confirms the coating of PVA on hydroxyapatite nanoparticles. The peaks corresponding to hydroxyapatite are 566, 602, 1029 cm^−1^, and peaks corresponding to PVA 1086, 1414 cm^−1^ are clearly visible. This confirms the coating of PVA on hydroxyapatite. The sustained release of amoxicillin from the nanocomposite is mainly due to the PVA coating on hydroxyapatite nanoparticles.

**Fig. 6:**
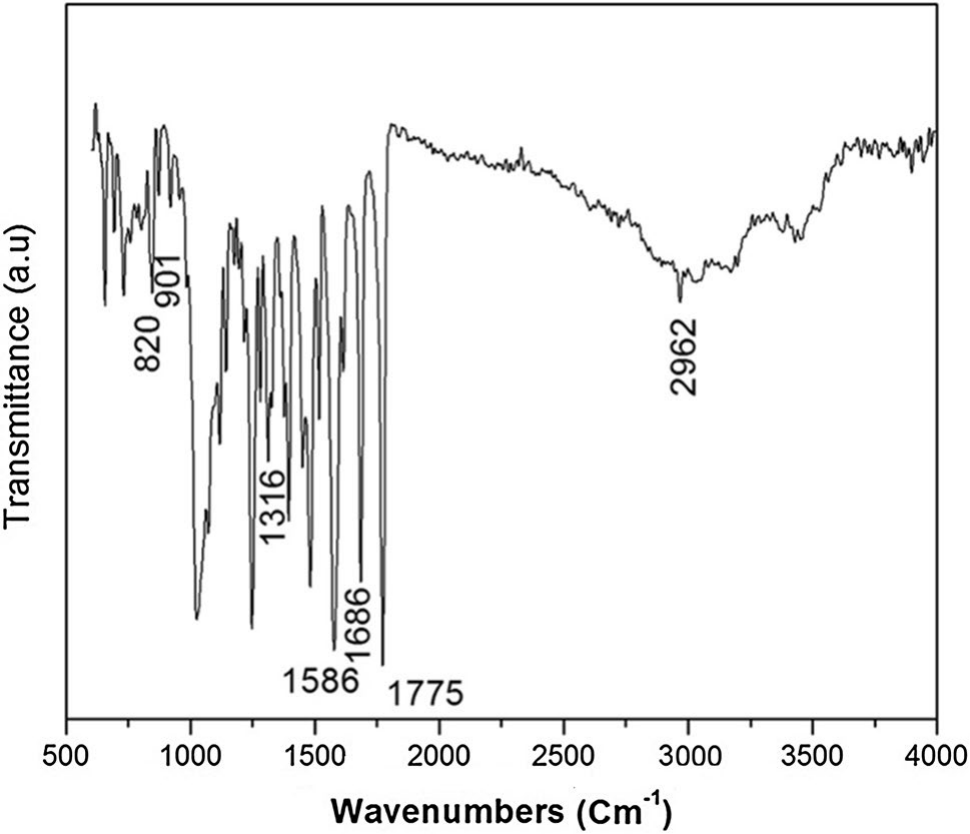
FTIR spectrum of HAP/PVA/SA/amox nanocomposite

The FTIR spectrum of HAP/PVA/SA/Amoxicillin is given in Fig. 6. Bands corresponding to the presence of calcium/Sodium alginate are present at 2962 cm^−1^, 1316 cm^−1^, 901 cm^−1^, 820 cm^−1^, 3445 cm^−1^, 1629 and 1418 cm^−1^. These bands are the combination of τCO, δCCO and δCCH. The peak broadening of *υ*_3_ PO^4^ is observed around 900–1200 cm^−1^. This confirms the presence of the polymer. The presence of amoxicillin is confirmed by the appearance of peaks at 1586, 1686 and 1775 cm^−1^. The encapsulation of drug is confirmed by FTIR analysis.

**Fig. 7:**
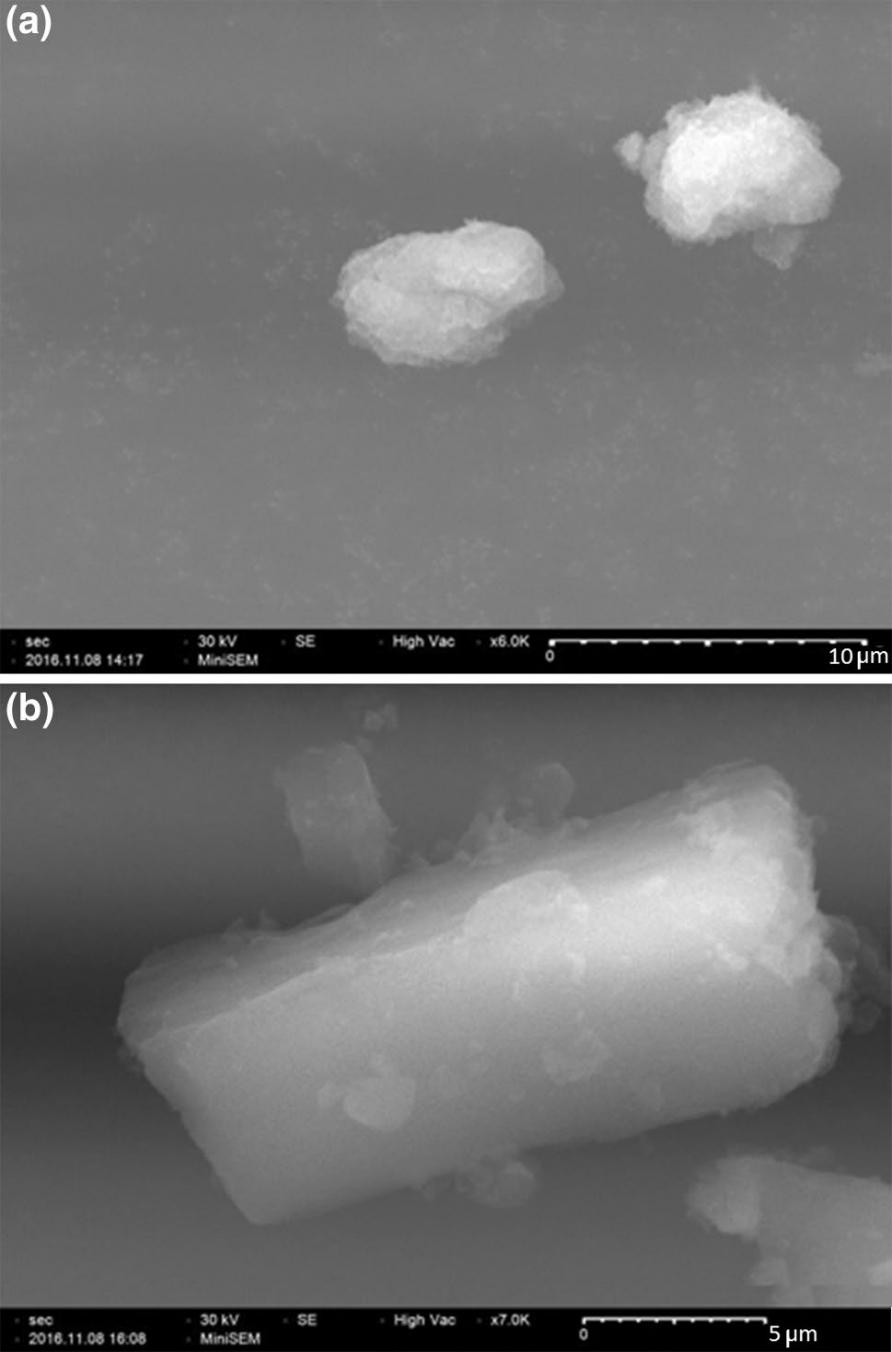
SEM image. **a** HAP/PVA nanocomposite **b** HAP/PVA/SA/amox nanocomposite

SEM image of the composite is given in Fig. 7. SEM image of HAP/PVA is given in Fig. 7a and image of HAP/PVA/SA/amoxicillin is given in Fig. 7b. PVA-coated hydroxyapatite nanoparticles are aggregated and aggregation is clearly visible in the image. The aggregation is due to the presence of PVA. Sodium alginate will form an outer layer on the composite. Drug would be entrapped in the polymer coating.

**Fig. 8:**
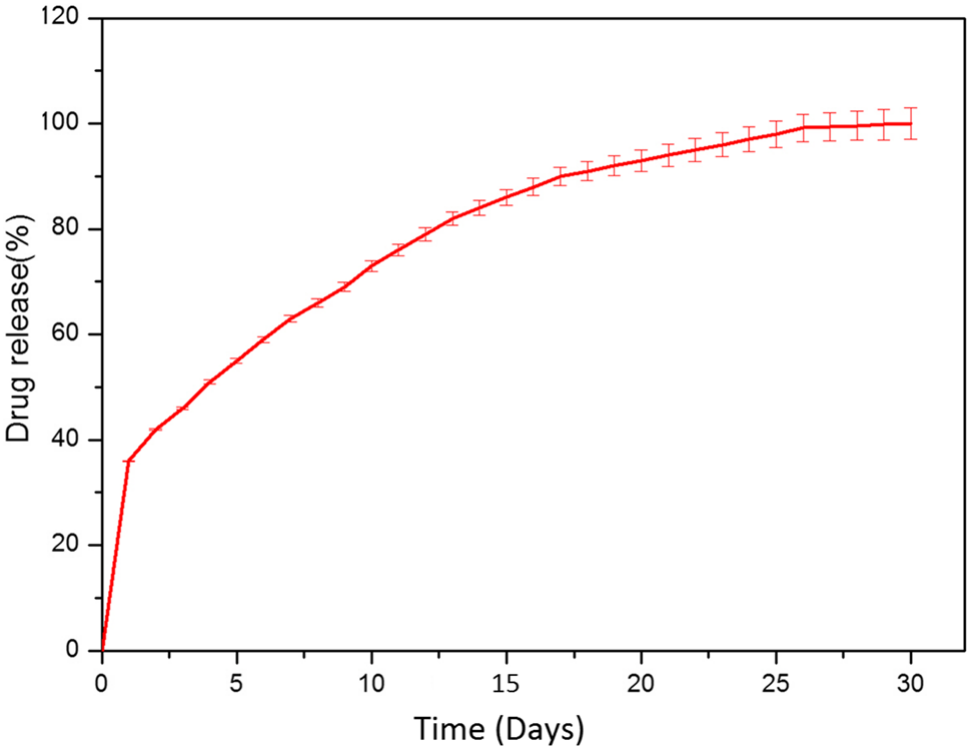
Drug release profile. Error bars represent calculations of standard error on the basis of triplicate determinations (mean ± SD, *n* = 3)

Figure 8 shows the drug release image of amoxicillin. The drug release was analyzed for a period of 30 days. The amoxicillin molecules present at the surface are released immediately. This leads to a sudden increase in the drug concentration. It is then followed by the degradation of sodium alginate layer on the surface of the nanoparticles. Sodium alginate will dissolve fast in the aqueous system than PVA. Polymer absorbs water and swells. When the polymer swells it begins to absorb more water. This will increase the pore size. Therefore, the drug is released through the pores. When the outer sodium alginate layer is fully degraded, then the PVA layer will start to absorb water. It will swell and the release of drug will increase. Since PVA has a low water absorbing capability at room temperature, the drug release will be slow and sustained. This makes this nanocompopsite suitable for the bone infections (Selvakumar et al. 2017). The sustained release of amoxicillin from the nanocomposite is similar to the membrane diffusion process. The amount of drug released on day 4 is 51%. This high release of drug is because of initial sudden burst and then due to the faster degradation of outer sodium alginate. Then, the drug release is very much sustained. The amount of drug released on day 10 is 73%, day 15 is 86% and day 20 is 93%. This is due to the slower degradation of PVA.

In the beginning, the drug release rate from nanocomposite is high and then it is decreased. The shrinking of HAP leads to the sudden increase in the concentration of the drug. The drug diffuses through the pores. The degradation of the polymer layer influences the drug release. The drug release depends on the dissolution of alginate and PVA. Amoxicillin is a hydrophilic drug. It absorbs water easily and diffuses through the pores in the polymer (Nakanishi et al. 2001).

**Fig. 9:**
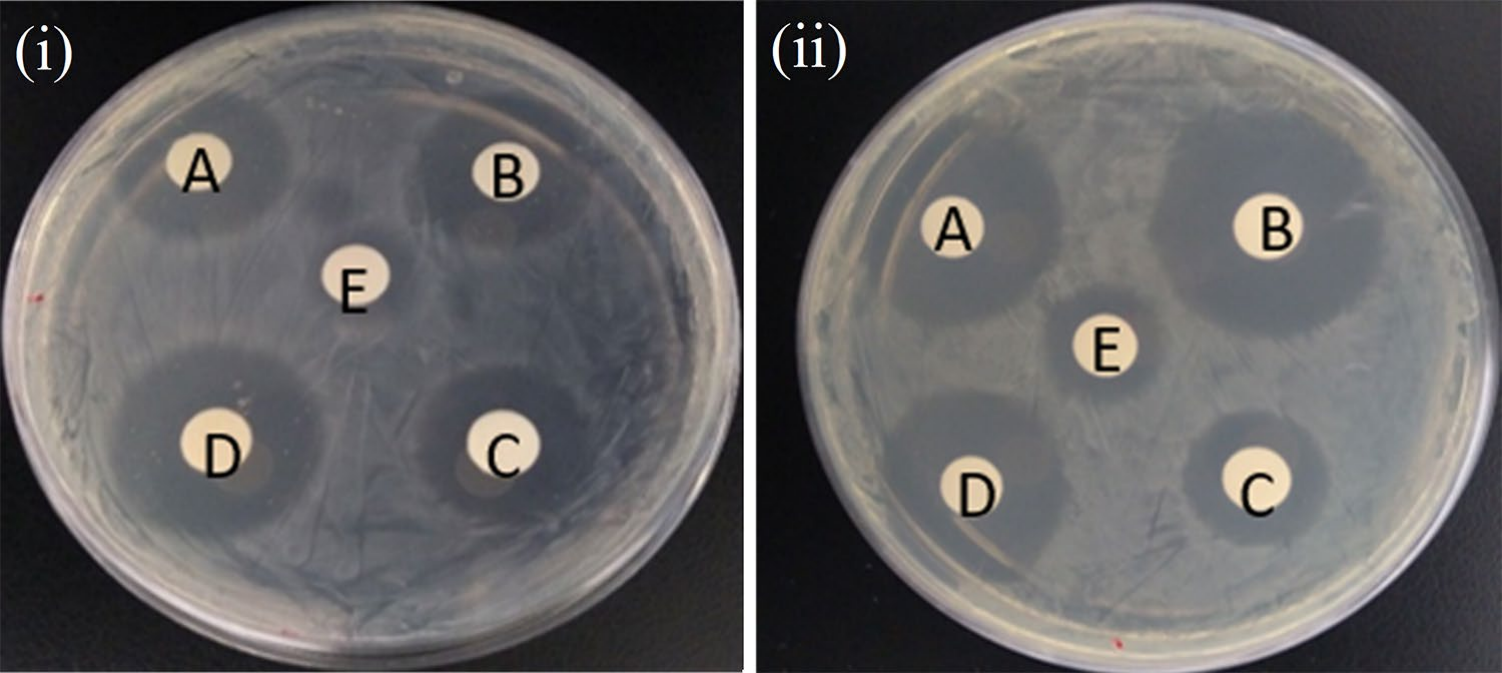
Antimicrobial activity of the nanocomposite against (1) *B. subtilis* (2) *K. pneumonia*. **a** 50 µg, **b** 100 µg, **c** 200 µg and **d** 500 µg

Antibacterial activity was done by well diffusion method against *Bacillus subtilis* and *Klebsiella pneumonia*. These two organisms are important because they cause bone infections. The inhibition zone against the two bone infections increases the pathogens with increases in concentration. This shows a very good antibacterial activity for the drug-loaded nanocomposites. The antibacterial activity against the pathogens is given in Fig. 9. The antibacterial activity against *Bacillus subtilis* is given in Fig. 9a and against *Klebsiella pneumonia* is given in Fig. 9b. The inhibition zones are seen very clearly. Hydroxyapatite nanoparticles will also exhibit antimicrobial effect (Baskar et al. 2017; Cui et al. 2016; Zhang et al. 2018). The inhibition zone is almost equal to the standard drug amoxicillin at lower concentration and it is higher than the standard drug at higher concentration (Table 1). This is because of the sustained release of drug from the nanocomposite. It is very difficult to cure bone infections, especially implant-associated infections. Antibiotics like amoxicillin have to be taken for a long duration. A sustained release of drug will be more effective in treating the bone infections. The sustained release of the drug from the nanocomposite exhibits an effective antibacterial activity. The layer-by-layer coating of polymers on the amoxicillin drug is very effective in the sustained release of amoxicillin.

**Table 1:** Zone of inhibition

Sample code	Zone of inhibition (mm)	
	*Bacillus subtilis* (mm)	*Klebsiella pneumoniae* (mm)
Amoxicillin (10 µg)	10	12
Nanocomposite (50 µg)	11	10
Nanocomposite (100 µg)	14	12
Nanocomposite (200 µg)	16	12
Nanocomposite (500 µg)	18	16

## Conclusion

Hydroxyapatite polymer nanocomposite was synthesized for sustained release of amoxicillin. PVA and sodium alginate are coated layer by layer on the hydroxyapatite nanoparticles. The layer by layer coating of polymers leads to the sustained release of amoxicillin. A sustained release of drug is observed from the nanocomposite for 30 days. The drug-loaded nanocomposite showed a very good antibacterial activity. Compared with the standard drug, the drug-loaded nanocomposite shows a good antibacterial activity. This ensures that this nanocomposite can be used as a drug delivery system for bone infections.
